# Aspect ratio dependent air stability of PbSe nanorods and photovoltaic applications

**DOI:** 10.3906/kim-2012-6

**Published:** 2021-06-30

**Authors:** Demet ASİL, Tuğba HACIEFENDİOĞLU

**Affiliations:** 1 Department of Chemistry, Faculty of Arts and Science, Middle East Technical University, Ankara Turkey; 2 The Center for Solar Energy Research and Application, Middle East Technical University, Ankara Turkey; 3 Department of Micro and Nanotechnology, Middle East Technical University, Ankara Turkey; 4 Department of Polymer Science and Technology, Middle East Technical University, Ankara Turkey

**Keywords:** Lead selenide, PbSe nanorods, PbSe quantum dots, stability, aspect ratio, solar cells

## Abstract

Development of unique strategies to overcome Shockley–Queisser (SQ) limit in solar cells has gained a great deal of interest. Multiple exciton generation (MEG) process has been considered as one of the best approaches to the SQ limitation. In this respect, PbSe quantum dots (QDs) and nanorods (NRs) have been regarded as promising solar energy harvesting materials owing to their noticeable MEG yields. Although air stability has been regarded as one of the main disadvantage of PbSe QDs, no study has pointed out to the air sensitivity of PbSe NRs yet. Here, we reveal the effect of aspect ratio on air sensitivity and optical properties of PbSe NRs and discover that NRs with higher aspect ratios are more air stable, attributed to the reduced density of NR ends with air sensitive {100} facets. Furthermore, a band offset was created by utilization of tetrabutylammonium iodide and 1,2-ethanedithiol ligands in cell designs. We found that solar cells based on pristine PbSe NRs are limited by low open circuit voltages due to leakage current pathways. On the other hand, modified cells comprising light absorbing layers prepared by blending NRs and QDs and hole transporting QD layer exhibit a 10-fold improvement in solar cell efficiency.

## 1. Introduction

Materials capable of harvesting solar energy and related technologies have attracted great deal of interest in recent years. Limitation of the ultimate photo conversion efficiency (PCE) of the crystal silicon solar cells to 33%, based on a study reported by Shockley and Queisser (SQ), led to the discoveries of new concepts that may be utilized to overcome the SQ limit [1]. Creation of multiple excitons with one photon absorption has been considered as one of the leading concepts in third generation solar cell field [2–5]. The presence of multiple excitation generation (MEG) process has been spectroscopically shown for various materials such as, nanoparticles, clusters and quantum dots [6–11]. Later, detailed studies on the effect of dimensionality on MEG yield have indicated that the MEG yield is more pronounced in elongated PbSe NRs compared to PbSe quantum dots [12]. In this sense, PbSe NR has been considered as one of the most promising materials to be utilized in third generation solar cells [5]. Although unique properties such as band gap (E_g_) tunability and higher MEG yield offer high promises for the lead based quantum dots to be utilized in future solar cell designs, their sensitivities toward ambient conditions limit their utilization in this field. In this sense, to improve their air stability and widen their application areas, various surface passivation techniques have been developed for extremely air sensitive PbSe and PbTe quantum dots [13–16]. Although many reports have pointed out to the effect of size and ligand type on the air sensitivity of QDs, no reports have described the effect of aspect ratio on NR stability yet [16–18]. In this study, we synthesized PbSe NRs with various aspect ratios and investigated their absorbance, photoluminescence and band alignment characteristics along with aspect ratio dependent air stability. Furthermore, solar cell characteristics of the PbSe NR based cells have been investigated and efficiency has been improved by 10-fold with the utilization of a thin hole transport layer at the absorbing layer-anode interface. 

## 2. Materials and methods

### 2.1. Materials

All chemicals were of the highest purity available unless otherwise noted and were used as received. All solvents were anhydrous and were used as received. Lead(II) oxide, (PbO, 99.999%, Aldrich, Saint Louis, MO, USA), selenium powder ( 200 mesh, 99.999%, Alfa Aesar, Ward Hill, MA, USA), oleic acid (OA, 90%, Aldrich), squalene (90%, Aldrich), cadmium chloride (CdCl_2_, 99.9%, Aldrich), tris(diethylamino)-phosphine (TDP, 90%, Aldrich), oleyamine (90%, Aldrich), benzene 1,3-dithiol (BDT, 99%, Aldrich), tetrabutylammonium iodide (TBAI, 99% Aldrich), titanium (IV) isopropoxide (TTIP, 99.999%, Aldrich), tetrachloroethylene (TCE, 99%, Aldrich), n-methylformamide (DMF, 99%, Aldrich), lead (II) acetate trihydrate (PbOAc,99.99%, Aldrich), 1-Octadecene (ODE, 90%, Aldrich), trioctylphosphine (TOP, 90%, Aldrich), diphenylphosphine (DPP,98%,Aldrich), 1,2-ethanedithiol (EDT, 98%, Aldrich), acetone (99.8%, Acros Organics), methanol (99.8%, Aldrich), buthanol (99.8%, Aldrich), hexane (99%, Aldrich), 2-propanol (99.5%, Aldrich), acetonitrile, (99.8%, Aldrich), octane (99%, Aldrich), tetradecylphosphonic acid (TDPA, 98%, Alfa Aeasar).

### 2.2. Synthesis of PbSe NRs

Synthesis of NRs was carried out following our previous reports [19]. All the NR formation reactions were performed using standard air-free Schlenk techniques and purifications were carried out in nitrogen filled glovebox (GB). For PbSe NRs with 1.0 eV band gap, PbO (1.31 g, 5.88 mmol) and OA (6 mL, 19 mmol) were mixed in squalene (30 mL). The reaction was kept under vacuum and N_2_ for about 4 h each. Se precursor was prepared by mixing TDP (18 mL, 65.7 mmol) and selenium powder (1.42 mg, 18 mmol) in GB. The injection was performed at 170 ºC. For PbSe QDs with 1.0 eV and 1.4 eV band gaps, PbOAc (1.30 g, 3. 44 mmol) and OA (2.68 mL, 8.4 mmol) were mixed in ODE (24 mL). The reaction was kept under vacuum and N_2_ for 4 h each. Se precursor was prepared by mixing TOP (10 mL, 22.4 mmol), selenium powder (0.789 mg, 10 mmol) and DPP (0.104 mL, 0.5976 mmol) in GB. The injection was performed at 145 ºC and 110.4 ºC for PbSe QDs with 1.0 eV and 1.4 eV band gaps, respectively. Purification and size-selective precipitation of the QDs (E_g_ = 1.4 eV), QDs (E_g_ = 1.0 eV) and NRs were done using hexane/methanol, hexane/acetonitrile and hexane/isopropanol/acetone solvent systems, respectively. The precipitated PbSe QDs and NRs were isolated by centrifugation (at 5000 rpm for 5 min) and dispersed in hexane. These steps were repeated three times. The final PbSe QDs and NRs were dispersed in octane for thin film applications and in TCE for characterization studies. All the samples for the related measurements were prepared using fresh nanoparticles.

### 2.3. Characterization of PbSe NRs

Solution (suspended in TCE) and thin film (on quartz spectrosils) UV-Vis-NIR spectra for PbSe NRs were recorded on a Shimadzu 3600 plus UV-Vis-NIR spectrometer. Photoluminescence (PL) measurements were performed by exciting the samples with a 980 nm laser diode. The PL emitted at a right angle relative to the excitation source was directed to a Newport emission monochromator and the PL signal was detected with a thermoelectrically cooled InGaAs photodiode (Hamamatsu-G6126). Corrected of the PL spectra were done by using the responsivity of the G6126 detector provided by the company. Transmission electron microscopy (TEM) samples were prepared by drop-casting a small volume of dilute PbSe NRs in TCE onto 200 Mesh, carbon coated copper TEM grids. After evaporation of the solvent at room temperature, a Jem Jeol 2100F 200kV HRTEM transmission electron microscope operating at 80–200 kV with Schottky type field emission gun as a source of electron was used for the TEM measurements. The CCD camera on which the images were recorded was GATAN, Orius SC10002. At least 140 NRs were examined by using ImageJ analysis program to determine the size distribution and the percentage of branching in PbSe NRs. The X-ray diffraction (XRD) measurements were performed on a high resolution Rigaku Ultima IV X-ray diffractometer with Cu X-Ray source, high resolution graphite monochromator and cross beam optics mechanism. For the XRD measurements, samples were prepared by spin casting of 25 mg/mL NRs solutions in octane onto a precleaned glass substrate.

Ultraviolet photoelectron spectroscopy (UPS) was used for the determination of the Fermi levels and valence band edges relative to vacuum. A PHI 5000 VersaProbe in ultrahigh vacuum conditions (10–10 mbar) with a monochromatic He I UV source (21.2 eV) was used for the measurements. A bias voltage of –7.00 V was used for the samples in order to separate the sample and the secondary edge for the analyzer and for the determination of the low energy cut-off. Thin films comprising PbSe NRs were prepared by spin coating onto Si/Cr (10 nm)/Au (150 nm) substrates in N_2_ filled GB and transferred to UPS set up with an air-tight tube. The samples were exposed to air only during loading to the instrument. Fermi level (reference to vacuum) was determined by the difference between the incident photon energy (21.2 eV) and high binding energy edge. Valance band energy minimum was determined from the low binding energy edge. Conduction band minimum was calculated from the subtraction of valance band minimum from the band gap. 

### 2.4. Solar cell fabrication and characterization

Indium tin oxide (ITO, Psiotec) patterned glass substrates were cleaned by sonication in acetone, isopropyl alcohol, hellmanex III and boiling deionized water for 15 min in sequence and dried in an oven at 100 ºC. The electron-transporting TiO_2_ layer was coated on ITO substrates after oxygen plasma etching of the cleaned ITO substrates for 15 min [16]. The titanium precursor was prepared by mixing solutions of TTIP (0.60 mmol, 175 µL) in 1.25 mL ethanol and addition of hydrochloric acid (2 M, 0.35 mmol, 17.5 µL) in 1.25 mL ethanol under vigorous stirring and filtered precursor mixture (0.45 PTFE) was spun on to ITO substrates at 5000 rpm for 30 s [20]. These sol-gel TiO_2_-covered substrates were annealed in air at 115 ºC for 30 min on a hot plate then 450 ºC for 30 min in a muffle furnace and left in air for a night. Active layers were deposited in a layer-by-layer (L-B-L) approach on top of TiO_2_ at 1500 rpm (2 s) and 2500 rpm (10 s) in the GB. NR/QD blends were prepared by mixing PbSe NRs (50 mg.ml^-1^) and PbSe QDs (50 mg.ml^-1^) in equal volumes. TBAI ligand exchange was done by applying a TBAI solution in methanol (50 mM) solution) to the surface of NRs (or blends) for 30 s. Excess ligands were removed by rinsing three times with methanol. For the EDT ligand exchange process, an EDT solution (0.02 vol% in acetonitrile) was spin coated on PbSe NRs and waited for 30 s for the complete ligand exchange of oleate ligands to EDT. Excess ligands were removed by rinsing three times with acetonitrile. Final washing step was performed by octane to remove excess NRs. For the TBAI treated layers, pristine PbSe NRs and blends with 25 mg mL^–^^1^concentration were used. On the other hand, for the EDT ligand exchanged layers, QDs (with 1.37 eV band gap) with 30 mg mL^–^^1^ concentration were utilized.Au (100 nm) metal contact was deposited by a thermal evaporator at a pressure of 10^–^^6 ^Torr and a rate of 0.1 Å/s through a shadow mask with an active area of 4.5 mm^2^. Finally, the cells were legged for electrical contacts and then encapsulated using transparent epoxy/resin and glass slides. 

The current-density-voltage characteristics were measured with a solar simulator (ASTM Class A stability) with AM1.5G sun irradiance (100 mW/cm^2^) over 50 × 50 mm area utilizing 100 W Xe arc lamp inside the GB at 25 ± 2 ºC and connected to a Keithley 2400 source measuring unit. A Newport calibrated reference solar cell (91150-KG5) where the certification has been accredited by NIST to the ISO-17025 standard was used for testing the photovoltaic cells under standard conditions. The devices were tested after the adjustment of the lamp power (after about an hour of warm-up time) level by the test cell until the irradiance level of the reference cell meter is set as 1.00 SUN. Results were averaged with a standard deviation across 4–8 cells. External quantum efficiency (EQE) measurements were performed outside (at 20–28 ºC with a 85 mm working distance) with a Newport QUANTX-300 apparatus. An Oriel monochromator (CS130) was used to filter out a narrow bandwidth of the incident white light source (100 W Xenon lamp) and tune its center wavelength across a desired range. The current generated by the test solar cell (603415 QUANTX-300) was compared to that generated by the calibrated photodiodes that have a known EQE. Silicon-germanium detector (PN: 603621) calibrated at NRC (Newport Research Center) within the accuracy specified by NIST (National Institute of Science and Technology) according to the 17025 certification standards was used as calibrated reference detector for 325–1800 nm range. EQE was measured with an uncertainty of ±25%, ±7% and ±3% for the wavelengths in between 300 and 330 nm, 330–340 nm and 350 nm–1800 nm, respectively.

## 3. Results and discussion

### 3.1. Aspect ratio dependent optical properties and air sensitivity

**Figure 1:**
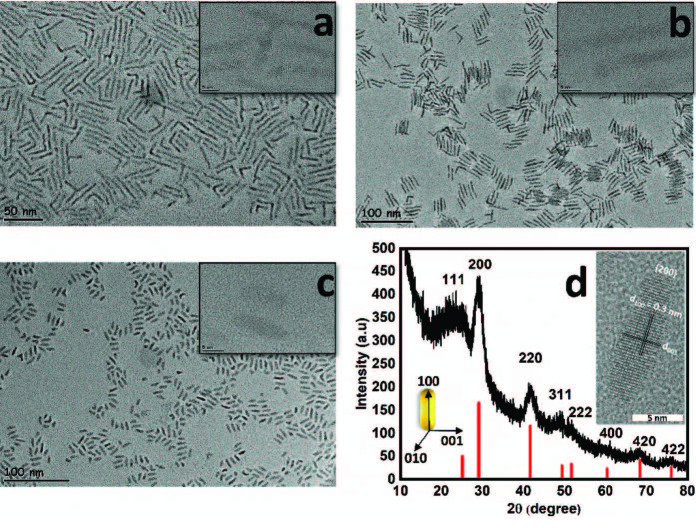
TEM images of PbSe NRs with aspect ratios a) 10.3 (scale bar: 50 nm), b) 8.3 (scale bar: 100 nm) and c) 3.0 (scale bar: 100 nm). Branching percentages were calculated as 37%, 12% and less than 1%. Inset: HRTEM images with a scale bar of 5 nm. d) Thin film XRD diffractions of BDT exchanged PbSe NRs on glass. XRD diffractions of PbSe QDs (JCPDS-ICDD card 06-0354) are shown (red) as a reference. Inset: HRTEM image (scale bar: 5 nm) representing fringe spacing of 0.30 nm.

To investigate the effect of aspect ratio on NR properties, a series of NRs with different length to diameter ratios was synthesized. Aspect ratios of the NRs were controlled by varying the stabilizing agent concentration and the injection temperature [19]. Figures 1a–1c present the high-resolution transmission electron microscopy (HRTEM) images of PbSe NRs with aspect ratios of 10.3, 8.3 and 3.0, respectively. High degree of crystallinity and homogeneity of PbSe NRs were proved by HRTEM measurements. Average diameters and lengths were analyzed by performing a size statistic on the HRTEM images and results are represented in Table 1. 

**Table 1:** Aspect ratio dependence optical properties of PbSe NRs.

ARa	Abs λmaxb	PL λmaxc	SSd	EGe	Df	L f	VBg	CBg	Shifth
3.0	1514 (140)	1540 (146)	26	0.81	4.1 ± 0.4	12.1 ± 2.3	–4.79	–3.98	48
8.3	1354 (156)	1440 (190)	86	0.89	3.2 ± 0.3	26.4 ± 4.7	–4.84	–3.95	11
10.3	1270 (160)	1350 (250)	80	0.95	3.1 ± 0.4	31.9 ± 7.1	–4.79	–3.84	6

We note a direct relationship with the percent branching and aspect ratio. As represented in Figure 1, PbSe NRs with 10.3 aspect ratio have a branching percentage of 37%, whereas the branching percentage drops to less than 1% as the aspect ratio is reduced to 3.0. This behavior was attributed to the lower concentration of the OA capping agents used for the synthesis of the shorter PbSe NRs [19,21]. HRTEM images of the individual rods represented as insets in Figure 1 indicate that the PbSe NRs have well-resolved lattice planes. As represented in Figure 1d-inset, PbSe NRs have a fringe spacing of 0.30 nm which corresponds to an interplanar distance of 0.30 nm, consistent with the (200) *d* -spacing of the PbSe cubic rock-salt structure. To investigate the crystallinity of PbSe NRs, X-ray scattering diffraction (XRD) measurement was performed on PbSe NRs. Figure 1d represents the diffraction peaks of PbSe NRs which are in good agreement with the PbSe QDs for (111), (200), (220), (331), (400), (420) and (422) without any impurity peaks (JCPDS-ICDD card 06-0354), suggesting a high degree of crystallinity and purity for the PbSe NRs utilized in this study. As reported previously, the growth direction of the PbSe NRs can be along {100}, {110} or {111} faces depending on the type and length of the stabilizing ligands used in the reaction [22,23]. According to previous reports, preferred alignment of NRs is expected to be along the 100 direction in the presence of short chain capping agents such as OA. As represented by the XRD pattern of the PbSe NRs in Figure 1d, enhanced (200) peak can be assigned to the preferred alignment of the PbSe NRs along the 100 direction. Therefore, the growth direction of PbSe NRs is along the [100] crystallographic axis which is parallel to the long axis of the rods.

**Figure 2:**
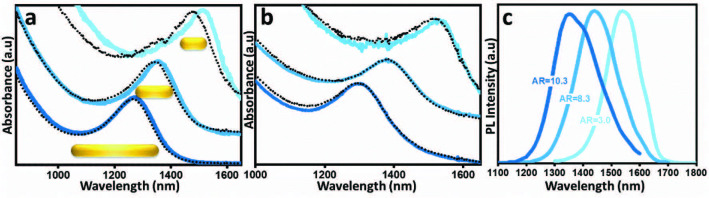
Optical properties of PbSe NRs. a) Solution and b) thin film absorbance spectra of PbSe NRs with aspect ratios 10.3, 8.3, and 3.0. PbSe NRs are dispersed in TCE and ligand exchanged with BDT for solution (a) and thin film (b) absorbance spectra, respectively. Dashed lines are representing the spectra measured after storing under ambient conditions for 12 days. c) Solution PL spectra of PbSe NRs (in TCE) with aspect ratios 10.3, 8.3 and 3.0.

To explore the optical properties of PbSe NRs with various aspect ratios, absorbance and photoluminescence (PL) measurements were performed in solution and on thin films. Featureless absorption and PL features presented in Figure 2 suggest that the PbSe NRs possess high monodispersity. Absorbance and photoluminescence spectra of the dilute solutions of PbSe NRs in TCE are represented in Figures 2a and 2c, respectively and optical properties are summarized in Table 1. Figure 2a represents the absorption spectra of the dilute solutions of the as synthesized OA-capped PbSe NRs with aspect ratios 3.0, 8.3, and 10.3. Absorbance peak points were recorded as 1514 nm, 1354 nm and 1270 nm for the PbSe NRs with aspect ratios 3.0, 8.3 and 10.3, respectively, whereas the corresponding PL peak points were measured as 1540, 1440 and 1350 nm. The absorbance spectra of BDT ligand exchanged PbSe NRs are represented in Figure 2b. The size, integrity and the absorption feature of the NRs are preserved when ligand exchanged with BDT. However, the excitonic peaks are red shifted in BDT exchanged close-packed NR thin films which can be attributed to the enhanced inter-NR electron coupling due to the reduced interparticle distances [24]. Previous reports have shown that the quantum confinement in anisotropic nanostructures is governed by the smallest length scale. In this sense, NR band gap shows a strong dependence on the NR diameter due to a significant influence of the lateral confinement on the band gap [25–28]. In line with those studies, the absorbance and PL peak points blue shift as the diameter of the NRs decreases. Stokes shift has been reported to be dependent on the NR energy fluctuations as a result of the length and diameter distribution [29]. In accordance with this study and as represented in Table 1, NRs with higher branching ratio exhibit higher FWHM as a result of energy fluctuations and show higher Stokes shift. As a result, NRs with lower aspect ratio can be synthesized with an improved branching ratio (and less FWHM) and show less Stokes shift as compared to NRs with higher aspect and branching ratio. The NR band gap energy, calculated from the position of the lowest energy 1S absorption feature of solution samples, is measured as 0.81 eV, 0.89 eV and 0.95 eV for the PbSe NRs with aspect ratios of 3.0, 8.3 and 10.3, respectively. 

To gain an insight on the effects of aspect ratio on the surface stability of PbSe NRs, thin films were exposed to air and the surface stability of PbSe NRs was assessed by measuring the shift in the absorbance peak wavelength of the PbSe NRs. It has previously shown that PbSe QDs exhibit surface oxidation and up to 50% of their volume is transformed into PbO, SeO_2_, or PbSeO_3_ within 24 h in ambient conditions [30]. Figures 2a and 2b show the change of the absorption peak for the NRs when stored under ambient conditions. According to the results indicated in Table 1, the first transition peak of the OA capped PbSe NRs in solution shows a blue shift of 48, 11 and 6 nm for the aspect ratios of 3.0, 8.3 and 10.3, respectively. Faster oxidation rate of the shorter PbSe NRs may be in relation to the {100}/{111} facet ratio which have known to have a significant effect on the surface stability [16]. Presence of excess Pb atoms on the {111} facets and higher affinity for ligand binding to the Pb atoms leads to a higher degree of protection on the surface Se atoms, which in turn suggests that {100} facets are more susceptible to oxidation due to higher degree of ligand loss [31]. In accordance with the enhanced (200) diffraction signal in XRD pattern, NRs align preferentially along the 100 direction which suggests that the NR ends are composed of 100 facets. It has previously been shown that the Se atoms on {100} facets are prone to oxidations whereas the oxidative attacks to Se atoms on the {111} facets are efficiently inhibited by the presence of excess Pb atoms on the {111} facets [31]. This suggests that the shorter NRs with higher ratio of {100} facets (NR ends) relative to stable {111} and {110} facets will lead to higher sensitivity towards oxidation due to less protected Se atoms on the surface. 

**Figure 3:**
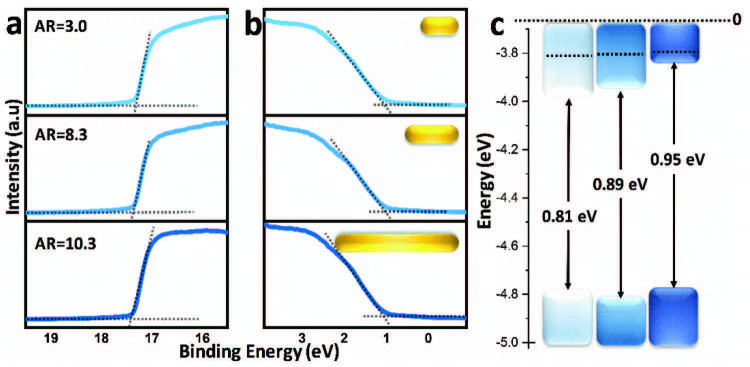
UPS spectra of PbSe NRs with aspect ratios 10.3, 8.3, and 3.0. a) High and b) low kinetic energy cut-off regions. Valence band energies with respect to vacuum were determined by the intersection of the linear fit of the main feature at high kinetic energy cut-off between the baseline. The work function was determined by extrapolating a linear fit of the low kinetic energy cut-off region to the xaxis. c) Energy landscape (relative to vacuum) of the PbSe NRs. Dashed lines represent the Fermi level.

We used ultraviolet photoelectron spectroscopy (UPS) to determine the effect of aspect ratio on PbSe NR band edge energies with respect to vacuum. Figure 3 shows the UPS spectra of PbSe NRs with aspect ratios 10.3, 8.3, and 3.0, respectively. According to the results summarized in Table 1, the valence band positions are not very sensitive to the aspect ratio of the NRs, whereas CB minimum shows a strong dependence on the aspect ratio. The CB minimum shifts closer to vacuum by up to 0.1 eV as the aspect ratio of the NRs increases, in line with theoretical studies reported before[27].

### 3.2. Solar cell characteristics of the PbSe NRs

**Figure 4:**
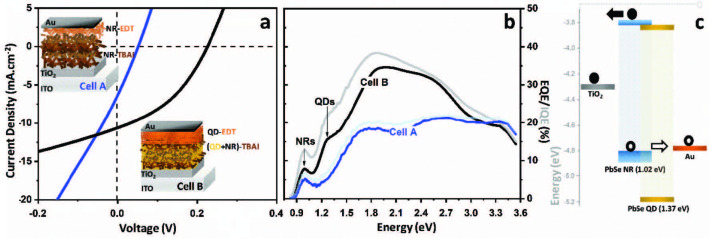
Solar cell characteristics of PbSe NRs. a) Current-voltage behavior of Cell A and Cell B. Inset: Schematic of Cell A: ITO/TiO2/NR-TBAI/NR-EDT/Au and Cell B: ITO/TiO2/(NR+QD)-TBAI/QD-EDT/Au. b) EQE and IQE of Cell A and Cell B. c) Energy landscape of the cell components referenced to vacuum level.

Previous studies have shown an enhanced PCE through band alignment engineering. Combining TBAI treated PbS QD layer with EDT treated QD layer created a band offset at the interface which facilitated the hole extraction while blocking the electron flow to the anode [32]. This approach of creating a band offset by applying different type of ligands to the QDs have been utilized as a key strategy to reach QD based solar cells with highest efficiencies [33–36]. We device a cell architecture (Cell A) using a similar strategy and treat the NRs with TBAI and EDT to create a band offset, as presented by the cell architecture in Figure 4a-inset. As indicated by the current-voltage characteristics in Figure 4a and summarized in Table 2, Cell A exhibits a short circuit current density (J_SC_) of 5.58 ± 0.59 mA cm^–^^2^ and open circuit voltage (V_OC_) of 0.04 ± 0.01 V. Cells based on pristine NRs showed a poor PCE of 0.055 ± 0.01% (see Table 2). We believe that the poor performance of NR based cells is due to an unoptimized thin film morphology of NR thin films [21]. To overcome morphology limitation, we modified the cell architecture by blending NRs (E_g_=1.02 eV) and QDs (E_g_=1.37 eV) in the same solution and used this blend during solid state ligand exchange with TBAI. The resulting thin films, composed of NRs and QDs linked by TBAI molecules, are expected to exhibit a bulk nano heterojunction (BNHJ) characteristics. Cell B, represented in Figure 4a, utilizes BNHJ films prepared by blending NRs and QDs with 1.02 eV and 1.37 eV band gaps, respectively. Additionally, a thin layer of EDT treated QDs with 1.37 eV band gap was used as a hole transporting layer between Au anode and BNHJ layer. As represented by the energy landscape in Figure 4c, lower valence band energy of PbSe QDs with 1.37 eV band gap allows efficient hole transport to the anode. Over 10-fold increase in PCE, compared to the cells based on pristine NRs (Cell A), was noted for the modified Cell B, as presented by the current-voltage diagram in Figure 4a. Modified cell yields J_SC_ of 10.34 ± 0.23 mA cm^–^^2^ and V_OC_ of 0.22 ± 0.01 V. Doubling of the photocurrent has been attributed to the extended interface between NRs and QDs which improves the electron transport by reducing the number of hopping events and efficient hole transport by the band offset provided by the QD layer. Improved charge transport is also confirmed by the higher shunt resistance (R_SH_) of the Cell B (R_SH_ =48.7 Ωcm^2^) compared to the Cell A (R_SH_ =9.23 Ωcm^2^). Lower R_SH _value for Cell A points out to the presence of alternate current paths for the light-generated current, reflecting the presence of current leakage paths and bimolecular recombination. Extremely low V_OC_ along with a significant power loss for Cell A was ascribed to lower R_SH_ as a result of the unoptimized thin film morphology of NRs. Enhanced charge transfer in Cell B was also confirmed by the EQE and internal quantum efficiency (IQE) measurements. Figure 4b presents the EQE and IQE spectra of Cell A and Cell B measured at short circuit conditions (zero applied bias). Theoretical J_SC_ values calculated by integrating the EQE spectra with the AM 1.5 G solar spectrum were 6.62 and 11.31 mA cm^–^^2^ for Cell A and Cell B, respectively, which are in line with the values obtained from current-voltage measurements. Presence of a good agreement in the short circuit current densities obtained from current-voltage and EQE setups validates the spectral measurements and enhances the reliability of the solar cell characterization. According to the EQE spectrum of the Cell A, photogeneration begins at 1.0 eV with a peak EQE of 5% and stays at 20% within 1.75 to 3.5 eV region. On the other hand, the peak EQE due to NRs in Cell B is enhanced to 8% at 1.0 eV, suggesting an enhanced charge generation in NRs or improved charge transport due to the formation of heterojunction at the QD-NR interface owing to BNHJ concept. An additional peak at 1.26 eV in EQE spectrum of the Cell B, which was absent in Cell A, was ascribed to the exciton generation by the QDs with 1.37 eV band gap, present in the BNHJ layer and in the hole transport layer. Higher exciton generation within a wide range of energies points out to the significance of BNHJ concept utilized in this study. 

**Table 2:** Solar cell characteristics of PbSe NRs.

	Jsca [mA.cm-2]	Voca [V]	FFa [%]	PCEa [%]	Jsc/EQEb [mA.cm-2]
Cell Ac	5.58 ± 0.59 (6.36)	0.04 ± 0.01 (0.06)	24.14 ± 3.67 (23.9)	0.055± 0.01 (0.085)	6.62
Cell Bd	10.34 ± 0.23 (10.62)	0.22 ± 0.01 (0.23)	37.10 ± 2.38 (37.38)	0.82 ± 0.04 (0.92)	11.31

## 4. Conclusion

In summary, we demonstrated PbSe NR based BNHJ type solar cells through engineering of the band alignment at the QD/NR and NR/anode interfaces, along with disclosure of the aspect ratio dependent air stability and optical properties. We discovered that the aspect ratio has a significant effect on air stability and attributed this behavior to the presence of air sensitive {100} facets located at the NR ends. Therefore, longer NRs with less number of {100} facets are more air stable compared to shorter NRs with more {100} facets. We also revealed that the valence band position is very sensitive to the aspect ratio, whereas no significant change has been noted for the position of the conduction band. We believe that the aspect ratio dependent air stability of nanorods described in this study may lead to the discovery of surface passivation techniques specially designed for NRs of desired length to diameter ratio. Furthermore, improved solar cell parameters due to the creation of band offset between NRs and QDs by forming a BNHJ and facilitation of the hole transport by the addition of a thin PbSe QD hole transport layer allowed us to fabricate solar cells with an enhanced PCE. We believe these results will lead to a greater understanding on the properties of PbSe NRs and further cell designs comprising morphology-controlled PbSe NRs will improve the MEG yield and enhance the solar cell efficiencies in near future. 
